# Lipoteichoic Acid Biosynthesis Inhibitors as Potent Inhibitors of *S. aureus* and *E. faecalis* Growth and Biofilm Formation

**DOI:** 10.3390/molecules25102277

**Published:** 2020-05-12

**Authors:** George A. Naclerio, Kenneth I. Onyedibe, Herman O. Sintim

**Affiliations:** 1 Chemistry Department, Institute for Drug Discovery, Purdue University, West Lafayette, IN 47907, USA; gnacleri@purdue.edu (G.A.N.); konyedib@purdue.edu (K.I.O.); 2 Purdue Institute for Inflammation, Immunology, and Infectious Diseases, West Lafayette, IN 47907, USA

**Keywords:** lipoteichoic acid inhibitor, wall teichoic acid inhibitor, methicillin-resistant *Staphylococcus aureus*, vancomycin-resistant *Enterococcus faecalis*, biofilm inhibition

## Abstract

Methicillin-resistant *Staphylococcus aureus* (MRSA) and vancomycin-resistant *Enterococcus faecalis* (VRE) have been deemed as serious threats by the CDC. Many chronic MRSA and VRE infections are due to biofilm formation. Biofilm are considered to be between 10–10,000 times more resistant to antibiotics, and therefore new chemical entities that inhibit and/or eradicate biofilm formation are needed. Teichoic acids, such as lipoteichoic acids (LTAs) and wall teichoic acids (WTAs), play pivotal roles in Gram-positive bacteria’s ability to grow, replicate, and form biofilms, making the inhibition of these teichoic acids a promising approach to fight infections by biofilm forming bacteria. Here, we describe the potent biofilm inhibition activity against MRSA and VRE biofilms by two LTA biosynthesis inhibitors HSGN-94 and HSGN-189 with MBICs as low as 0.0625 µg/mL against MRSA biofilms and 0.5 µg/mL against VRE biofilms. Additionally, both HSGN-94 and HSGN-189 were shown to potently synergize with the WTA inhibitor Tunicamycin in inhibiting MRSA and VRE biofilm formation.

## 1. Introduction

Antimicrobial-resistant bacteria have become a serious global health issue. The World Health Organization (WHO) acknowledges that every year 700,000 people die from drug-resistant infections worldwide. It has been estimated that deaths from drug-resistant infections will reach 10 million people per year by 2050, surpassing deaths due to cancer [1]. The Centers for Disease Control and Prevention (CDC, USA) has reported that on average two million people are inflicted with an antibiotic-resistant infection every year, and at least 23,000 people die from these infections [2]. Of these drug-resistant bacteria, methicillin-resistant *Staphylococcus aureus* (MRSA) and vancomycin-resistant *Enterococcus faecalis* (VRE) are recognized as serious threats by the CDC. MRSA accounts for over 80,000 infections and over 11,000 deaths annually while VRE accounts for about 20,000 infections and 1300 deaths per year [2].

The majority of chronic MRSA and VRE infections are due to biofilm formation. Biofilm is a group of bacterial pathogens that anchors to a biological (lung, intestine, tooth) or non-biological (medical devices) surface and biofilm bacteria are 10–1000 times more resistant to antibiotics than planktonic bacteria [3]. Currently, treatment for MRSA and VRE biofilm infections involves long-term antibiotic therapy, which leads to increased persistence and destruction of inflamed tissue [4]. Thus, new agents that eradicate or inhibit MRSA and VRE biofilm formation via novel mechanisms are needed.

**Figure 1:**
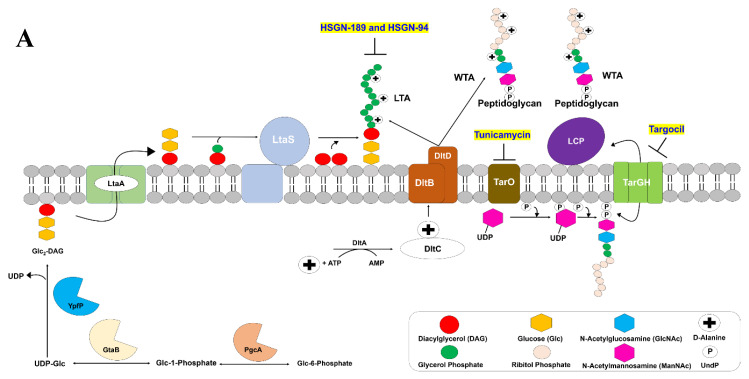

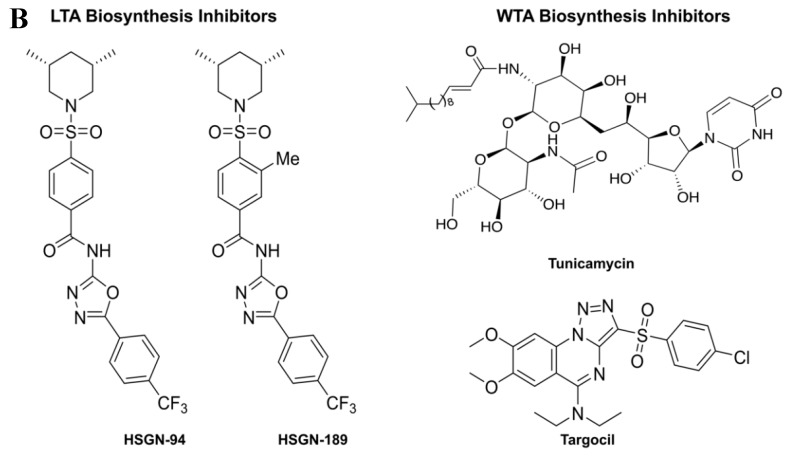
(**A**) LTA biosynthesis occurs at the Gram-positive bacterial cell membrane. The α-phosphoglucomutase PgcA converts glucose-6-phosphate to glucose-1-phosphate, then uridyltransferase GtaB activates uridine triphosphate (UTP) to produce UDP-glc. Glc_2_-DAG is then produced from YpfP transfering two glucose molecules from UDP-Glc to DAG. Glc_2_-DAG is moved to the outer membrane by LtaA followed by LtaS adding glycerol phosphate to Glc_2_-DAG generate LTA. WTA biosynthesis begins in the cytoplasm where TarO plays a key role in generate the diphospho-ManNAc-GlcNAc-GroP polymer. TarGH then exports the WTA polymer to the cell membrane where the LytR-CpsA-Psr (LCP) proteins catalyze the covalent bond between the WTA and peptidoglycan. The d-alanine moieties are added by DltABC. (**B**) HSGN-94 and HSGN-189 inhibit LTA biosynthesis. Tunicamycin and Targocil inhibit WTA biosynthesis via inhibition of TarO and TarGH, respectively.

Teichoic acids are abundant throughout the cell envelopes of Gram-positive bacterial pathogens such as *S. aureus*, enterococci, *Listeria monocytogenes*, *Streptococcus pneumoniae*, and *Bacillus subtilis* [5]. Teichoic acids are divided into two classes: lipoteichoic acids (LTAs) and wall teichoic acids (WTAs) (Figure 1A). Both LTA and WTA play major roles in Gram-positive bacterial cell processes that are vital to their survival [5]. Specifically, LTA is an anionic 1,3-glycerolphosphate containing polymer anchored to the cell wall while WTA is a cell surface glycopolymer that is covalently linked to peptidoglycan and expands beyond the cell wall [6,7]. Both LTA and WTA are very important for bacterial growth, cell wall physiology, membrane homeostasis, and virulence [8]. Regarding biofilm formation, both LTA and WTA are vital. For instance, teichoic acids lacking d-alanine showed decreased colonization of both MRSA and VRE, as well as reduced adherence of these bacterial pathogens to nasal epithelial cells [9,10,11]. Both LTA’s and WTA’s important roles in biofilm formation have been linked to disruption of the negative charge of the bacterial cell wall resulting in altered hydrophobicity [12]. Therefore, both LTA and WTA can be potential targets in the development for new antibacterial agents against biofilm forming Gram-positive infections.

WTA inhibitors have been developed [13,14]. Tunicamycin, a natural product, is an inhibitor of TarO, a biocatalyst in the first step of WTA biosynthesis (Figure 1). Likewise, the novel antibiotic Targocil, inhibits TarG, a main component of the ABC transporter TarGH (Figure 1) [13,15]. Both Tunicamycin and Targocil possess antibiofilm activities as well as potentiate the effects of other antibiotics [13,14,16].

Very few LTA biosynthesis inhibitors exist [17,18]. Recently, we reported novel *N*-(1,3,4-oxadiazol-2-yl)benzamide containing LTA biosynthesis inhibitors with MIC values as low as 0.25 µg/mL and 1 µg/mL against MRSA and VRE, respectively (Figure 1) [19,20]. In this follow-up study, we sought to determine the activity of our two most potent LTA biosynthesis inhibitors, HSGN-94 and HSGN-189, against MRSA and VRE biofilm formation. Here, we report HSGN-94 and HSGN-189 as having potent biofilm inhibition activity against MRSA and VRE with minimum biofilm inhibition concentrations (MBICs) as low as 0.0625 µg/mL and 0.5 µg/mL, respectively. Additionally, HSGN-94 and HSGN-189 showed potent synergism or additivity when tested in combination with Tunicamycin and Targocil against MRSA and VRE planktonic bacteria and biofilms.

## 2. Results and Discussion

### 2.1. Biofilm Inhibition Activity of HSGN-94 and HSGN-189 against MRSA and VRE Strains

**Figure 2:**
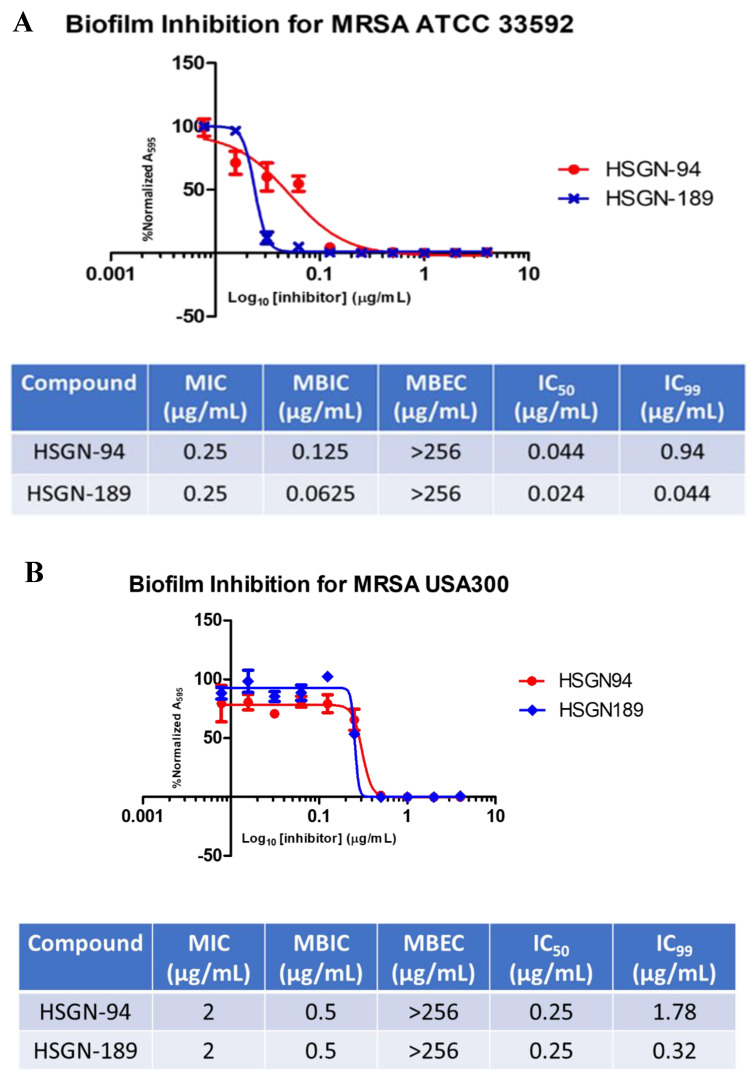

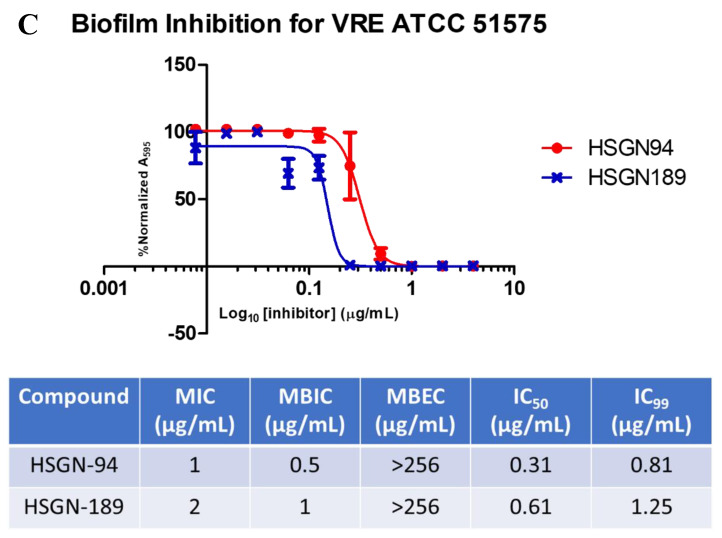
(**A**) Biofilm Inhibition Curves for HSGN-94 and HSGN-189 against MRSA ATCC 33592. (**B**) Biofilm Inhibition Curves for HSGN-94 and HSGN-189 against MRSA USA300. (**C**) Biofilm Inhibition Curves for HSGN-94 and HSGN-189.

The synthesis and characterization of both HSGN-94 and HSGN-189 have been previously described [19]. Additionally, HSGN-94 and HSGN-189 were found to have potent antibacterial activity against both MRSA and VRE with MICs as low as 0.25 µg/mL and 1 µg/mL, respectively. Furthermore, both compounds proved to be the most potent LTA biosynthesis inhibitors published [19]. As mentioned above, since LTA plays a major role in biofilm formation of both MRSA and VRE, we aimed to test whether HSGN-94 or HSGN-189 could have antibiofilm activity. Both HSGN-94 and HSGN-189 showed potent biofilm formation inhibition against MRSA and VRE with minimum biofilm formation inhibition concentrations (MBICs) at or below their MIC values. For instance, the MBIC of HSGN-94 against MRSA ATCC 33592, MRSA USA300, and VRE ATCC 51575 was found to be 0.125 µg/mL, 0.5 µg/mL, and 0.5 µg/mL, respectively (compare with MICs of HSGN-94 to these strains being 0.25 µg/mL, 2 µg/mL, and 1 µg/mL, respectively; see Figure 2). Similarly, HSGN-189 also had potent MBIC values against MRSA ATCC 33592, MRSA USA300, and VRE ATCC 51575 and was found to be 0.0625 µg/mL, 0.5 µg/mL, and 1 µg/mL, respectively, which are all below the reported MIC values (see Figure 2). Both HSGN-94 and HSGN-189 did not disperse established biofilms. Since the MBIC values of the compounds are lower than MIC (for example HSGN-189 inhibits biofilm formation of MRSA ATCC 33592 at a concentration that is 4× lower than MIC (MIC = 0.25 µg/mL and MBIC is 0.0625 µg/mL), we conclude that the mode of biofilm inhibition is not entirely due to bacterial death. We do not discount that some bacterial death also account for biofilm formation inhibition since at the MBIC concentrations, some bacterial death (not 100%) was also observed, see Figure 2A,C. Thus, it appears that although LTA is critical for initial biofilm formation, other factors are also important for biofilm maturation and persistence [21,22]. Established biofilms contain many adhesive and connective compounds, including DNA, proteins, and polysaccharide [23,24,25,26]. Thus, agents that degrade these would also be needed to eliminate established biofilms. In any case, combining LTA and WTA inhibitors with biofilm degraders, such as proteases [27], DNAses [28,29], and β-hexosaminidases [30,31] could lead to enhanced biofilm clearance and worthy of future investigations.

### 2.2. HSGN-94 and HSGN-189 Synergize with Tunicamycin and Targocil against MRSA and VRE Strains

Tunicamycin’s and Targocil’s effect on WTA biosynthesis has been linked to their ability to synergize with cell-wall targeting antibiotics [15,32]. For instance, Tunicamycin was shown to synergize with β-lactam containing antibiotics such as cefotaxime, ceftazidime, methicillin, oxacillin, and cephradine; Tunicamycin enhanced the activities of these antibiotics by 4 to 64 times [15]. Similarly, Targocil was also tested in combination with representative antibiotics of different classes but only synergized with methicillin (the cell-wall targeting antibiotic) with a ∑FICI of 0.4 [32]. Considering that HSGN-94 and HSGN-189 act on the cell-wall via inhibition of LTA biosynthesis, we wondered if our compounds would be synergistic with Targocil or Tunicamycin against MRSA and VRE. Using the checkerboard assay described below, we probed interactions between HSGN-94 and HSGN-189 in combination with WTA inhibitors against drug resistant MRSA ATCC 33592, MRSA USA300, and VRE ATCC 51575 strains (Table 1).

HSGN-94 or HSGN-189 in combination with Targocil resulted in additivity or indifference for all three strains. Combining HSGN-94 with Targocil against MRSA ATCC 33592 resulted in an eight-fold decrease in MIC for Targocil, from 16 µg/mL to 2 µg/mL while also decreasing the MIC for HSGN-94 from 0.5 µg/mL to 0.25 µg/mL (Table 1A). Although, either HSGN-94 or HSGN-189 did not show significant synergy in combination with Targocil, there was remarkable reduction in Targocil’s MIC against MRSA USA300 or VRE, from >1024 µg/mL to 16 µg/mL (approximately a hundred-fold decrease in Targocil’s MIC) (see Table 1B,C). However, HSGN-94 in combination with Tunicamycin resulted in synergy against MRSA ATCC 33592 and VRE ATCC 51575. Against MRSA ATCC 33592, Tunicamycin’s MIC decreased from 256 µg/mL to 64 µg/mL while HSGN-94′s MIC went from 0.5 µg/mL to 0.125 µg/mL (Table 1A). Against VRE ATCC 51575, Tunicamycin’s MIC went from 16 µg/mL to 4 µg/mL, resulting in a 4-fold change (Table 1C). Combining HSGN-94 with Tunicamycin against MRSA USA300 resulted in additivity with Tunicamycin with a 16-fold change in MIC (Table 1B). Likewise, combinations with HSGN-189 and Tunicamycin resulted in synergy when tested against MRSA USA300 and VRE ATCC 51575. Against MRSA USA300, synergy between HSGN-189 and Tunicamycin resulted in a 16-fold decrease in MIC for Tunicamycin (Table 1B). Similarly, for VRE ATCC 51575, combinations with HSGN-189 and Tunicamycin resulted in Tunicamycin’s MIC decreasing from 16 µg/mL all the way down to 0.5 µg/mL (Table 1C). Against MRSA ATCC 33592, combinations between HSGN-189 and Tunicamycin resulted in additivity with Tunicamycin experiencing an 8-fold change in MIC (Table 1A).

### 2.3. HSGN-94 and HSGN-189 Shows Synergy with Tunicamycin in Inhibiting MRSA and VRE Biofilms

Tunicamycin has been previously reported to inhibit *S. aureus* and *L. monocytogenes* biofilm formation. Since HSGN-94 and HSGN-189 showed synergistic activity with Tunicamycin, we sought to determine if these compounds could synergize with Tunicamycin to inhibit MRSA and VRE biofilms. Thus, following a previously reported procedure [33], we determined the MBIC values of HSGN-94 and HSGN-189 in combination with Tunicamycin against clinically relevant MRSA USA300 and VRE ATCC 51575 biofilms. Interestingly, both HSGN-94 and HSGN-189 showed synergy with Tunicamycin in inhibiting MRSA USA300 and VRE biofilm formation. Alone, the MBIC of Tunicamycin was found to be 64 µg/mL against MRSA USA300 biofilms but, in combination with HSGN-94, the MBIC of Tunicamycin decreased 32-fold to 2 µg/mL, resulting in a ∑FICI of 0.5 (Table 2A). HSGN-94 also showed potent synergy with Tunicamycin against inhibiting VRE biofilms (Table 2B). Additionally, HSGN-189 showed synergy with Tunicamycin against MRSA USA300 biofilm formation. Tunicamycin’s MBIC went from 64 µg/mL to 4 µg/mL when combined with HSGN-189 (Table 2A). Furthermore, combinations with HSGN-189 and Tunicamycin resulted in synergism in inhibiting VRE ATCC 51575 biofilm formation resulting in a ∑FICI of 0.3 (Table 2B).

## 3. Materials and Methods

### 3.1. Bacterial Strains and Chemical Compounds

Bacterial strains used in this study were obtained from the American Type Culture Collection (ATCC). Tunicamycin and Targocil were purchased from Cayman Chemical (Cayman Chemical Company, Ann Arbor, MI, USA). HSGN-94 and HSGN-189 were previously synthesized from commercial sources in our laboratory.

### 3.2. Synergistic Interactions of HSGN-94 and HSGN-189 with Tunicamycin and Targocil

The checkerboard assay [34,35] was used to determine synergistic interactions of antibiotic-compound combinations against MRSA ATCC 33592, MRSA USA300, and VRE ATCC 51575. Tunicamycin and Targocil were tested in combination with compounds HSGN-94 or HSGN-189. The ΣFICI was calculated for each combination as follows:

FICI _compound_ = MIC of HSGN-94 or HSGN-189 in combination/MIC of HSGN-94 or HSGN-189 alone (1)

FICI _antibiotic_ = MIC of antibiotic in combination/MIC of antibiotic alone (2)

The cumulative FICI (∑FICI) was then calculated as:

∑FICI = FICI _compound_ + FICI _antibiotic_ (3)

Interactions where the ΣFICI was ≤0.5 were categorized as synergistic (SYN). An ΣFICI of >0.5–1.25 was categorized as additive (ADD). ΣFICI of >1.25–4 was considered as indifference (IND), while ΣFICI values of >4 were categorized as antagonistic [36].

### 3.3. Biofilm Inhibition Assay and Minimum Biofilm Inhibition Concentration (MBIC)

MRSA and VRE biofilm inhibition were performed in tissue culture treated 96 well plates. Overnight cultures of MRSA ATCC 33592, MRSA USA300, and VRE ATCC 51575 were diluted 1:100 in tryptic soy broth (TSB) supplemented with 1% glucose. The diluted culture was inoculated into wells with 1 mg/mL stock solution of compound in DMSO (at 4 μg/mL to 0.0078 μg/mL). DMSO contents ranged from 0.8% (in the 4 μg/mL well) to 0.002% (in the 0.0078 μg/mL well). The growth control did not contain any compound. The sterility control contained only media (TSB supplemented with 1% glucose). The plates were incubated at 37 °C for 48 h after which the minimum biofilm inhibition concentration (MBIC) was read as the minimum concentration of the compounds that completely inhibited the visual growth of biofilm. Next, medium was carefully discarded, and the unattached cells washed away. The biofilms were stained with 0.5% crystal violet for 30 min. The crystal violet was discarded, and wells washed. The dye was solubilized with 100% ethanol for 1 h and the biofilm mass was quantified by measuring absorbance at 595 nm on a BioTek Cytation 5 Cell Imaging Multi-Mode Reader (BioTek, Winooski, VT, USA). The A595 value for any absorbance reading, *A*, was normalized to the no compound (*A*_T_) and broth (*A*_o_) controls using the Equation (4):

(4) $$\%\;Normalized\;A595=\left(\frac{A-A_{0}}{A_{r}-A_{0}}\right)\times 100$$

### 3.4. Biofilm Eradication Assay and Minimum Biofilm Eradication Concentration (MBEC)

MRSA and VRE biofilm eradication were performed in tissue culture treated 96 well plates. Overnight cultures of MRSA ATCC 33592, MRSA USA300, and VRE ATCC 51575 were diluted 1:100 in tryptic soy broth (TSB) supplemented with 1% glucose and further incubated to OD_600_ 0.2. Next, the culture was diluted 1:10 in TSB supplemented with 1% glucose and inoculated into wells. The plates were incubated at 37 °C for 24 h. Then, the medium was carefully discarded, and the unattached cells washed away. Compound (at 256 μg/mL to 0.5 μg/mL) in TSB supplemented with 1% glucose was added to the preformed biofilm. The plates were incubated at 37 °C for 24 h after which the minimum biofilm eradication concentration (MBEC) was read as the minimum concentration of the compounds that completely eradicated the preformed biofilm. Next, medium was carefully discarded, and the unattached cells washed away. The biofilms were stained with 0.5% crystal violet for 30 min. The crystal violet was discarded, and wells washed. The dye was solubilized with 100% ethanol for 1 h and the biofilm mass was quantified by measuring absorbance at 595 nm on a BioTek Cytation 5 Cell Imaging Multi-Mode Reader (BioTek, Winooski, VT, USA). The A595 value for any absorbance reading, *A*, was normalized to the no compound (*A*_T_) and broth (*A*_o_) controls using Equation (4).

### 3.5. MBIC Synergy with Tunicamycin

The checkerboard assay was utilized as described above. However, tryptic soy broth (TSB) supplemented with 1% glucose was used as the primary medium and the plates were incubated at 37 °C for 48 h. After, the medium was discarded, and the unattached cells washed away. The biofilms were stained with 0.5% crystal violet for 30 min. The crystal violet was discarded, and wells washed. The ΣFICI was calculated for each combination as follows:

FICI _compound_ = MBIC of HSGN-94 or HSGN-189 in combination/MBIC of HSGN-94 or HSGN-189 alone (5)

FICI _antibiotic_ = MBIC of antibiotic in combination/MBIC of antibiotic alone (6)

The cumulative FICI (∑FICI) was then calculated as:

∑FICI = FICI _compound_ + FICI _antibiotic_ (7)

Interactions where the ΣFICI was ≤0.5 were categorized as synergistic (SYN). An ΣFICI of >0.5–1.25 was categorized as additive (ADD). ΣFICI of >1.25–4 was considered as indifference (IND), while ΣFICI values of >4 were categorized as antagonistic [36].

## 4. Conclusions

We previously identified HSGN-94 and HSGN-189 as novel LTA biosynthesis inhibitors. Here, we demonstrate that these compounds have potent inhibition of MRSA and VRE biofilms with MBICs well below compounds’ MICs. Additionally, these compounds showed synergistic activity when combined with WTA inhibitors Tunicamycin and Targocil. Furthermore, HSGN-94 and HSGN-189 also showed potent synergy with Tunicamycin in inhibiting MRSA and VRE biofilms significantly decreasing the MBIC of Tunicamycin from 64 µg/mL to 2 µg/mL against MRSA. Therefore, we demonstrate that potent inhibitors of LTA biosynthesis (such as HSGN-94 and HSGN-189) can be used to inhibit biofilm infections from Gram-postive bacterial pathogens, either alone or in combination with WTA inhibitors. Opoku-Temeng et al. reported that compounds containing the *N*-(1,3,4-oxadiazol-2-yl)benzamide moiety, as found in HSGN-189 and HSGN-94, were efficacious in vivo and reduced bacterial load in a mouse wound infection model. Future work will be focused on making HSGN-94/189 analogs thereof and evaluate these compounds in vivo [20].

## Figures and Tables

**Table 1:** (**A**) The cumulative fractional inhibitory concentration index (∑FICI) range of HSGN-94 and HSGN-189 in combination with Tunicamycin and Targocil against MRSA ATCC 33592. (**B**) The cumulative fractional inhibitory concentration index (∑FICI) range of HSGN-94 and HSGN-189 in combination with Tunicamycin and Targocil against MRSA USA300. (**C**) The cumulative fractional inhibitory concentration index (∑FICI) range of HSGN-94 and HSGN-189 in combination with Tunicamycin and Targocil against VRE ATCC 51575. Note: ∑FICI was interpreted as follows: ∑FICI of ≤0.5 is considered to demonstrate synergy (SYN). An ΣFICI of >0.5–1.25 was categorized as additive (ADD). ΣFICI of >1.25–4 was considered as indifference (IND), while ΣFICI values of >4 were categorized as antagonistic.

A	MRSA ATCC 33592											
	MIC Alone		Combination MIC		∑FICI	SYN/ADD/IND	MIC Alone		Combination MIC		∑FICI	SYN/ADD/IND
Antibiotic	Antibiotic	HSGN-94	Antibiotic	HSGN-94			Antibiotic	HSGN-189	Antibiotic	HSGN-189		
Targocil	16	0.5	2	0.25	0.6	ADD	32	0.5	16	0.5	1.5	IND
Tunicamycin	256	0.5	64	0.125	0.5	SYN	256	0.5	32	0.25	0.6	ADD
**B**	**MRSA USA300**											
	**MIC Alone**		**Combination MIC**		**∑FICI**	**SYN/ADD/IND**	**MIC Alone**		**Combination MIC**		**∑FICI**	**SYN/ADD/IND**
**Antibiotic**	**Antibiotic**	**HSGN-94**	**Antibiotic**	**HSGN-94**			**Antibiotic**	**HSGN-189**	**Antibiotic**	**HSGN-189**		
Targocil	>1024	2	16	2	1.0	ADD	>1024	2	16	2	1.0	ADD
Tunicamycin	32	2	2	1	0.6	ADD	64	2	4	0.5	0.3	SYN
**C**	**VRE Faecalis ATCC 51575**											
	**MIC Alone**		**Combination MIC**		**∑FICI**	**SYN/ADD/IND**	**MIC Alone**		**Combination MIC**		**∑FICI**	**SYN/ADD/IND**
**Antibiotic**	**Antibiotic**	**HSGN-94**	**Antibiotic**	**HSGN-94**			**Antibiotic**	**HSGN-189**	**Antibiotic**	**HSGN-189**		
Targocil	>1024	2	16	2	1.0	ADD	>1024	2	16	2	1.0	IND
Tunicamycin	16	2	4	0.5	0.5	SYN	16	2	0.5	1	0.5	SYN

**Table 2:** (**A**) MBIC of HSGN-94 and HSGN-189 in combination with Tunicamycin against MRSA USA300 biofilms. (**B**) MBIC of HSGN-94 and HSGN-189 in combination with Tunicamycin against VRE ATCC 51575 biofilms. ∑FICI was calculated and interpreted as follows: ∑FICI of ≤0.5 is considered to demonstrate synergy (SYN). An ΣFICI of >0.5–1.25 was categorized as additive (ADD). ΣFICI of >1.25–4 was considered as indifference (IND), while ΣFICI values of >4 were categorized as antagonistic.

A	MRSA USA300											
	MBIC Alone		Combination MBIC		∑FICI	SYN/ADD/IND	MBIC Alone		Combination MBIC		∑FICI	SYN/ADD/IND
Antibiotic	Antibiotic	HSGN-94	Antibiotic	HSGN-94			Antibiotic	HSGN-189	Antibiotic	HSGN-189		
Tunicamycin	64	2	2	1	0.5	SYN	64	2	4	0.5	0.3	SYN
**B**	**VRE Faecalis ATCC 51575**											
	**MBIC Alone**		**Combination MBIC**		**∑FICI**	**SYN/ADD/IND**	**MBIC Alone**		**Combination MBIC**		**∑FICI**	**SYN/ADD/IND**
**Antibiotic**	**Antibiotic**	**HSGN-94**	**Antibiotic**	**HSGN-94**			**Antibiotic**	**HSGN-189**	**Antibiotic**	**HSGN-189**		
Tunicamycin	32	2	8	0.06	0.3	SYN	32	2	8	0.06	0.3	SYN
